# Polygenic risk for schizophrenia predicts dopamine‐related grey matter volume alterations in people with Alzheimer’s disease and psychotic symptoms

**DOI:** 10.1002/alz.086510

**Published:** 2025-01-09

**Authors:** Riccardo Manca, Annalena Venneri

**Affiliations:** ^1^ Brunel University London, Uxbridge UK; ^2^ Brunel University London, London UK; ^3^ University of Parma, Parma Italy

## Abstract

**Background:**

Psychotic symptoms may manifest in Alzheimer’s disease (AD), especially in advanced disease stages and in patients with higher polygenic risk scores for schizophrenia (SCZ‐PRS). Such genetic risk seems also to influence grey matter volume (GMV) alterations in patients with psychosis. Since multiple neurotransmitter systems, namely dopamine (DA) and serotonin (5‐HT), have been implicated in psychosis, the aim of this study was to investigate whether a SCZ‐PRS may explain variance in the association between GMV and the cerebral distribution of DA and 5‐HT.

**Method:**

Eight‐hundred ADNI participants with genetic data were selected for this study: 203 cognitively unimpaired (CU) and 597 people with AD. Patient were divided into psychotic (PT‐PS, n = 121) and non‐psychotic (PT‐NP, n = 476) based on available NPI‐Q data. A SCZ‐PRS was calculated for each participant using a Bayesian approach. T1‐weighted MRI scans were pre‐processed with SMP12 and JuSpace to extract Fisher’s z‐transformed individual correlation coefficients between GMV and PET atlases for DA and 5‐HT receptors/transporters. General linear models were used to test the association between the SCZ‐PRS and GMV‐neurotransmitter correlation coefficients (p < 0.05). Analyses were replicated in a sub‐sample of amyloid‐positive participants.

**Result:**

The SCZ‐PRS was negatively associated with correlations coefficients between GMV and the D2 dopaminergic receptor (D2r) in the whole sample (β = ‐0.016, p = 0.023) and in the PT‐PS group (β = ‐0.037, p = 0.030). However, the latter association was significant only when analyses were restricted to amyloid‐positive participants (Figure 1). In the PT‐NP group, the SCZ‐PRS was negatively associated with correlations coefficients between GMV and two 5‐HT receptors (5‐HT_1b_: β = ‐0.014, p = 0.027; 5‐HT_2a_: β = ‐0.013, p = 0.038). No between‐group differences in SCZ‐PRSs were found.

**Conclusion:**

The SCZ‐PRS was differentially associated with GMV alterations linked to either DA, in PT‐PS, or 5‐HT, in PT‐NP, that are both neurotransmitters previously implicated in psychotic symptoms. However, the influence of SCZ‐PRS on DA‐related GMV loss appears to be primarily relevant to psychotic manifestations in AD. These findings suggest that future innovative pharmacological interventions targeting the DA system may be beneficial to treat psychosis in AD.

